# Horseshoeing—A Criticism*An answer was hurriedly written at the time and sent to the *Herald*, but rejected.

**Published:** 1892-05

**Authors:** James Hamill

**Affiliations:** 347 E. 14th St., New York


### HORSESHOEING—A CRITICISM. *

By James Hamill, V. S.

An article of some importance to the veterinary profession appeared in the *New York Herald* of April 3d, headed, "Worth Knowing About Shoeing." The alleged statements of Mr. Robert Bonner in regard to the horse's foot and its treatment by the shoeing-smith and veterinary surgeon, strike at both parties with a force most remarkable, even for Mr. Bonner.

While I am in accord with the general drift of the subject, I think a few of the statements might be modified considerably without injury to, or detracting from the usefulness or meaning of what has been said, because every sentence spoken or written by that distinguished gentleman regarding the horse, and especially concerning the foot, is accepted by the public—and with good reason—as from a very high authority, and therefore mistakes should be corrected. I admire Mr. Bonner for his superior knowledge of all that pertains to the trotting horse, and also for the possession of all the qualities and principles that make the *man*, but I stand ready to oppose him when he says, "that the blacksmiths of to-day have caused more actual misery than all the burglars and all the murderers!" I have never had a very high opinion of the shoeing-smiths as a class, but I do think, that even in New York city, they will compare favorably with any other men who are compelled to do the very hardest labor. Their intelligence may not be of the highest order, as the reward in horseshoeing is not enough to induce graduates of Yale and Princeton to take up the business as a profession. Were a shoeing-smith possessed of manhood, the learning, scientific attainments and experience requisite to be as perfect as Mr. Bonner's ideal workman, he would very soon tire of the business, because he would find that he could earn fifteen to twenty-five thousand dollars per year at something else, while shoeing would only yield him a part, and probably small part of that amount.

Much of the ignorance and degradation that Mr. Bonner charges against the shoeing-smiths may be traced to the horse-owners themselves. The majority of the horse-owners are in the habit of sending their horses to the shoeing forge in charge of the

* An answer was hurriedly written at the time and sent to the *Herald*, but rejected.

very worst element of "stable help," whose aim is to teach the shoer how to shoe the foot, etc., rather than deliver the few brief orders of "the boss." Sometimes, indeed, they are "loaded" with authority, and often with beer, and then "they know everything about the horse, and particularly about his feet and shoeing." I have often wondered why the average smith should waste valuable years of his life learning how to shoe, when, at a moment's notice, he has every horseman, every coachman, driver, groom, and stable boy, aye, and every bummer who is able to stagger to the shop with a horse to be shod, to tell him how to do his work on that and every other horse ; nothing is left to the judgment of the man who has probably been thirty to forty years in practice.

Does this state of affairs exist in any other branch of business ? I think not, as I have never seen those vampires, which are allowed about many stables, take their employer's gold chronometer watches to the watchmaker to be repaired, and there dictate the details of the work; yet a good horse has a superior value to a watch.

Mr. Bonner's arraignment of the veterinary profession is on a par with that of the shoers. If they are as ignorant as he claims—and he makes no distinction—the Society for the Prevention of Cruelty to Animals should institute legal proceedings against all of them.

No one can deny that the blistering of tendons and ligaments that are sprained, while the cause was neglected, would be cruelty. Yes, it would be cruelty indeed, and the perpetrator should be punished severely. But the veterinary profession, I presume, like every other profession, is made up of individuals differing very much in character and ability, yet it is an axiom in their practice "*to remove the cause before the result.*" There is but one grand exception in veterinary practice, and I can hardly see why any man would be more blind to *cause* than *effect*, from the fact that he was a veterinary surgeon.

The keeping of the "feet level" is an old story, and has been the hobby—and a proper one—of authorities on the foot before Mr. Bonner was born. The question of how to level it is still an open one, as the mere leveling of the shoe to fit the foot, or of the foot to fit the shoe, and the juxtaposition of two surfaces is no proof that either is level. However, "*keep the feet level*" is sound advice, and should be carried out consistently with the superstructure, so that the results looked for may be achieved. And all that can be expected, all that can be desired, by any and every system of shoeing, can be stated in a few words, viz.: *Freedom of action and security of foothold.* The condensed "lore" of all the horsemen in the world is in that one short sentence.

A long experience in practice does not confirm Mr. Bonner's theory "that it is absolutely impossible for a horse to throw out a spavin unless he has a long toe."

He does not say anything about the results of *concussion.* Many other statements are, to say the least, very misleading. It is admitted that the hoof must be trimmed, but then "there are nerves in it;" no sane man would touch the nerves of a horse's foot before he was thoroughly secured, and we should be told where the nerves are situated and where they terminate. He says: "Now, as to the dreaded navicular disease. No man can tell, or ever will be able to tell whether a horse has navicular disease or not, unless he dissects the foot." To my mind, Mr. Bonner has a good deal to learn. In navicular disease, there are times when it would be difficult to make a decisive diagnosis; but when certain stages are reached, the expert surgeon can detect it as easily as he would any other morbid condition of the limb or foot, without dissecting it.

Then there is that "sweeping statement," "upon which the veterinary authorities unanimously agree, and upon which they are all entirely wrong," and including Professor Williams. The statement is that if you raise the heels of a horse's shoes, you raise the animal's ankle (fetlock), and if you raise the toe you depress the ankle. Mr. Bonner says: "I have proved that it's wrong over and over again." I have had the pleasure of seeing Mr. Bonner trying to prove this, about fifteen years ago, when he enjoined silence on the part of a few friends, to whom he explained his discovery, and all have honorably kept their word since. But as he has been the first to make it public, there can be no objection to open the discussion now. The veterinary profession are right. And Professor Williams is correct, although some of the woodcuts which illustrate the subject in his text book do not do him justice.

Mr. Bonner is very much in error in regard to the effect on the fetlock joint of a high-heel shoe. A high heel does tend to raise the fetlock joint and produce a columnar position of the pastern bones. This can be easily demonstrated on almost any horse. Of course there are some exceptions where we meet with long flexor tendons and weak suspensary ligaments, but all raising of the heels relieves the flexor tendons of the foot and also the suspensary ligaments from tension. I agree with Mr. Bouner that while doing so the heel must be forced toward the fetlock, or more properly toward the bones of the pastern. Where he makes the serious blunder—and it's a pity—is in using only the dry bones of the foot for illustrating the point. He ignores the function of the tendons and ligaments of the part. He ignores every other tissue which aids in forming and supporting the joints of the foot; in fact, he ignores every physiological condition of the limb and foot, and therefore deceives himself, while the fact can be proven in a few minutes on the living horse, and by the merest novice among horsemen. But Mr. Bonner, like a modern Samson, crushes the entire veterinary profession with the bones. I endorse Mr. Bonner's views regarding the diploma for the shoeing-smith, before he is allowed to practice on our dumb beasts, and also State supervision of examinations for both shoers and veterinary surgeons, so that no unfit person would be granted a license. And I'm sure Mr. Bonner was right in withholding his check on the occasion mentioned in the *Herald*, such a stand is just the thing to spur the schools to better work, as none of the veterinary schools in existence have ever done their part properly. In the interest of the horse, and to give a fair hearing to Mr. Bonner, the shooing-smith and veterinary surgeon, I will undertake to give a lecture or number of lectures on the anatomy and physiology of the horse's foot and its relation to shoeing. Lectures to be illustrated as circumstances will permit of, and as they should be given at the veterinary schools. Mr. Bonner and such friends as he may select to have every opportunity for discussion, and if he is agreeable I shall have arrangements made free to all. I have no ulterior designs, only to defend a much abused profession, as according to an old saying "a certain character is not as black as he is painted.

With feelings of due respect for Mr. Bonner, I remain,

Yours truly,

347 E. 14TH ST., NEW YORK. JAMES HAMILL, V. S.

